# Supplementary material for: Determinants of formula feeding among mothers with infants and young children in six Sub Sahara African countries: Multilevel analysis of data from demographic and health survey
Source: PLoS One. 2024 Dec 26;19(12):e0311945. doi: 10.1371/journal.pone.0311945 (PMC11670973; doi:10.1371/journal.pone.0311945)
Supplement: Attachment — Submitted filename: Point by point response to reviewers.docx [file pone.0311945.s003.docx]

**Point by point response to reviewers’ comments**

Dear Editor and Reviewers, thank you very much for your valuable and thoughtful comments, suggestions, and recommendations. We have accepted and appreciated all your comments and suggestions to our manuscript. We have addressed all the comments by providing point by point response and correction to the manuscript. Your comments are crucial to produce high quality paper. We have significantly revised and improved the quality of the manuscript guided by your comments, suggestion, and recommendations. Here below are presented the responses of your comments for each point.

**Reviewer #1**: Thank you for reviewing the DHS findings from six countries. Some of your conclusions appear to be misleading, as statistical significance does not always align with real-world outcomes. For instance, factors such as maternal education, institutional delivery, and exposure to media were identified as significantly linked to bottle feeding. However, is this truly justifiable in practical terms, especially considering our emphasis on promoting and prioritizing institutional deliveries?
**Author’s response:** We have accepted your valuable comments; we have made correction and amendments to the manuscript, thank you very much dear reviewer.

Maternal education, institutional delivery, and media exposure are statistically linked to bottle feeding. However, practical justifiability depends on context. Here’s why:

1. **Maternal Education**: Higher education often correlates with better health knowledge. Educated mothers may make informed feeding choices. Yet, practicality varies—some educated mothers may still opt for formula due to personal reasons or cultural norms.
2. **Institutional Delivery**: Promoting institutional deliveries is crucial for maternal and neonatal health. However, the link to bottle feeding isn’t straightforward. Institutional births don’t guarantee exclusive breastfeeding. Postpartum support and counseling matter.
3. **Media Exposure**: Media influences behavior. But practicality differs. Exposure to formula ads might sway some mothers, while others prioritize breastfeeding despite media messages.

In the discussion section, while you have referenced supportive articles to support your findings, it would be prudent to also include articles that present contrary evidence against your statistical conclusions.

**Author’s response:** Considered, thank you

**Reviewer #2:** The authors have analyzed the key factors associated with formula feeding among mothers with infants in six sub-Saharan African (SSA) countries. The data for the analyses come from the MEASURE Demographic and Health Survey program - specifically the Kids Record (KR) subset. The authors have performed multi-level multivariable analysis to identify the determinant factors at both individual and community levels.
As a reviewer, I did not have a hard time reading the manuscript or understanding the sequence of arguments presented in (a) justifying the necessity of such a study, and (b) substantiating the model choices and the results thereafter. However, in a major portion of the manuscript, the arguments themselves are inadequate, and the analyses seem almost mechanical and brute-force, allowing little room for data exploration and judgments that is a fundamental part of any statistical analysis workflow.
As such, the manuscript is problematic in its current form for me in both methodological and scientific ways, and I hope my point-by-point queries presented below will help the authors to formulate the paper in an improved, sound fashion.

**Author’s response:** We have accepted your valuable comments that are helpful to improve the quality of the manuscript; we have made correction and amendments to all of your concerns, thank you very much dear reviewer.

1. The authors summarize the study variables in Tables 1, 2, and 3, but spend very little time in trying to assess what those percentages mean. Lines 176-196 very briefly touch upon some of these percentages, but fail to mention some key aspects.

**Author’s response:** We have considered it and made amendments in the manuscript. Lines 176-196 in the result section are descriptive for the socio-demographic and economic variables presented in frequency and percentages. Moreover, the important aspects are presented in the discussion and conclusion section of the manuscript. Your comments are crucial, thank you very much dear reviewer.

2. 1.a. There are some categorical variables that are extremely imbalanced - such as child plurality (almost a 96 to 4 breakup). Are such variables even fit to be included in any model?

**Author’s response:** Child plurality is categorized as single childbirth and twin childbirth. The commonest one is single childbirth twin pregnancy is rare and triple and more pregnancy almost none. It fulfills the Chi-square assumption. So, like any other variables, we can test for the association with the outcome variable because we have no option other than this, thank you very much dear reviewer.

3. 1.b. The authors absolutely ignore the fact that the overall summaries in Tables 1-2 do not represent any potential country-specific pattern. What if a certain country has higher or lower proportions of a particular age group of mothers, or differences across education or marital status? Do we know that all SSA countries are uniform in these terms? Only a country-specific summary of each variable will answer this - and only after that we can even talk about which variables are worth including in any candidate model at all.

**Author’s response:** That is impressive comment. All SSA countries are not uniform in their characteristics. We used the appended data (merged data) from six countries. So, it is difficult to describe country specific details. It needs another study Of course; each country did not have the same sample size. Whatever the sample size in each country, the main thing is that this study was based on the data collected by probability sampling techniques throughout the whole areas of each country. This probability based survey study was presented in percentage. So, it is representative for the general population. We have considered your comment and made some amendments in the manuscript, thank you so much.

4. The modeling scheme seems rushed and in some places, odd. There are quite important questions that the authors do not even attempt to answer.

**Author’s response:** We appreciate your comment; we have made correction and amendments to all of your concerns, thank you very much dear reviewer.

5. Table 3 clearly shows that formula feeding itself is not consistent across the countries. Gabon is a huge outlier. Ghana and Somalia are similar, with Cameroon and Ethiopia showing much smaller percentages. It is extremely possible that many of the associations identified in the models are country-specific variations in demographics and health indicators masked as true determinants. Whether there is country-specific effect modification or confounding of any kind is a question that needs to be answered before making any kind of conclusive statement.

**Author’s response:** That is great insight, thank you very much dear reviewer. Every country has its own proportion of formula feeding practice. Dear reviewer, please consider that there is no countries having similar proportion of formula feeding practice in Africa. Each countries proportion of FF is different. Your question is nice but it will be answered by country specific study. The country specific –variability is already known. Our study is about merged data of six SSA countries to assess the overall burden and its determents of FF.

6. The authors mention that variables with a p-value < 0.2 in a bivariate analysis were included in the multivariate models. In the three non-null models, individual and community-level variables are included and assessed in an all-or-nothing fashion. I do not see the point of this. A logical way to build a 'best' model would be to have the variables with p < 0.2 as potential candidates, and then use a forward selection in the null model or backward selection in model iii to choose the best set of variables. Things like size of child at birth and age of child are nowhere significant - why should a so-called 'best' model include them? Statistically, a 'best' model should cut down as much slack as possible without removing true associations.

**Author’s response:** The best fit model is a model having highest log-likelihood ratio (LLR) and lowest deviance that is model III, which is fitted by including all variables at the same time both individual and community level variables. No matter any variable can be included in best fit model because the variables fulfill Chi-square assumption and described in adjusted odd ration (AOR) after adjusting the potential confounding factor. In addition, as you said, we have also used a logical way to build a 'best' model would be to have the variables with p < 0.2 as potential variables. Thank you very much for your nice recommendation dear.

7. All the models are simple association models - that too with data from different years for different countries. There is no temporal factor, nor any possibility of inferring any kind of causality from such data. As such, statements like "maternal age affects infant feeding practices" (line 279) may be true, but are beyond the scope of this work. The only thing that can be said here is something in the lines of what the authors say immediately after this - "Those mothers older than 25 years were more likely to utilize formula feeding as compared to those younger." All statements in the discussion section should avoid using terms like affect/impact or likewise. Non-causal statements maintained by association and likelihood are the only possible inferences here.

**Author’s response:** Thank you very much, we appreciate your impressive comments, simply accept your comment and have made correction.

8. A huge component of the analysis is constituted by the sample weights that the authors mention in many places but never provide any information on.

**Author’s response:** Sample weights are essential for valid and meaningful analyses in DHS surveys. The purpose of sample weight includes

- To make sample data representative of the entire population.
- To account for differential probabilities of selection.
- To correct for nonresponse and adjust for complex survey design.
- To avoid missed observations

Thank you very much

9. How are these weights constructed? Are they provided as part of the original survey dataset? If yes, what methods did they use to arrive at these weights?

**Author’s response:** The sample weight was carried out by using the formula on the STATA software. “gen wgt= v005/1000000.” “tab outcome[iw=wgt]”

We have considered your comments, thank you very much dear reviewer.

10. The entire point of using survey weights is to adjust sample contributions in the perfect way. In this scenario, a sample may amount to more than a sample size of 1 if it is deemed to be substantially different from other data points. On the other hand, 10 very similar samples may end up being treated like a single sample if they are too similar or correlated. This begs the question: are the summaries in Tables 1-3 absolute summaries of the observations or are these weights taken into account there as well?

**Author’s response:** We used the weighted sample to compensate for the unequal probability of selection between the strata and to keep the sample representative. In the data analysis and descriptive summaries, we have considered and used the weighted data. Thank you very much dear reviewer.

11. What is the purpose of Figures 1-2? They do not provide any new information beyond the summary tables.

**Author’s response:** [Of course you are right, but to provide more description on our results, we think that figures have visual impact and effectively communicate our primary findings](https://writingcenter.unc.edu/tips-and-tools/figures-and-charts/). They allow us to display information easily, thank you very much.

**Reviewer #3:** Dear Author,
Thank you for the nice work. The discussion section is shallow and please discuss in detail with available published data elsewhere. Please update old reference. The rest of this work I found as very interesting

**Author’s response:** Thank you very much dear reviewer, we appreciate your valuable comments that are crucial to improve the quality of the manuscript; we have made correction and amendments to all of your concerns.

**Reviewer #4:** Thank you for doing this research in this area. It was well done and written with some minor corrections in a few sections of the paper. I have included the comments I have in the document attached, please.

**Author’s response:** We appreciate your impressive and attractive comments that are important to improve the quality of the manuscript; we have made correction and amendments to all of your concerns, thank you very much dear reviewer.

**Reviewer #5:** This paper addresses an important topic, but I have some concerns about this study entitled “Determinants of formula feeding among mothers with infants and young children in six Sub-Saharan African countries: Multilevel analysis of data from demographic and health survey.”

**Author’s response:** We appreciate your valuable comments that are important to improve the quality of the manuscript; we have made correction and amendments to all of your concerns, thank you very much dear reviewer.

1. In the abstract part of the manuscript line 31 “the prevalence of formula feeding among mothers with infants was 17.1%.” it is better to say the proportion of mothers with infants who use formula feeding was 17.1%."

**Author’s response:** Corrected, thank you very much dear reviewer.

2. In line 35 “higher odds was observed among plurality of child (AOR = 1.4; 95% CI (1.23-1.77))” What mean a plurality of children? Better to use the term Multiple Children.

**Author’s response:** Plurality of child means “single or twin” childbirth; we have made correction, thank you very much

3. In lines “26 &27” The data were taken from a recent demographic and health survey in six sub-Sahara African countries. Why only six countries? What is the reason behind that?

**Author’s response:** The burden of the problem assumed to be high in those countries included in this study than any other SSA countries from reviewing literature, the aim of the study was to assess this burden and its determinants, thank you very much

4. Given that more than six countries have conducted similar surveys after 2019, the focus on only six countries might limit the generalizability of the findings.

**Author’s response:** We have confirmed that there might be related studies but there is no similar study about formula feeding and its determinant factors in SSA, thank you very much dear reviewer.

5. The conclusion: the conclusion lacks clarity and specificity: specify why formula feeding is a concern (e.g., health risks, economic factors, cultural issues). Additionally, lacks consistency and uniformity: make sure the suggestions make sense in light of the identified issue.

**Author’s response:** We have considered in the manuscript, thank you very much.

6. Furthermore, it has to be implementable suggestions: Make clear the steps that policymakers and stakeholders should take.

**Author’s response:** We have considered in the manuscript, thank you very much.

7. In the discussion section line 265 -266, the prevalence of formula feeding might be lower in large-sample studies than in basic studies, do you have pieces of evidence? If you have evidence it must be cited

**Author’s response:** Yes, the prevalence of formula feeding can appear lower in large-sample studies due to better statistical precision, reduced bias, and increased representativeness. We have cited the evidence on the manuscript. Thank you very much.

8. The discussion section especially for the second objective is shallow and lacks appropriate citations.

**Author’s response:** We considered, thank you very much dear reviewer.

9. Consider starting with a summary of your main findings before diving into specific associations.

**Author’s response:** We have considered in the discussion line 274-278, thank you

10. In lines 301-305 Maintain consistent terminology when referring to groups. For example, use "educated mothers" and "mothers with no formal education" consistently throughout the section. Please clearly state the observed association and provide potential reasons supported by the literature.

**Author’s response:** We have corrected and made amendments on the discussion section of the manuscript based on your recommendation, thank you very much dear reviewer.

11. Why do you fail to explain why community literacy levels might impact individual feeding practices?

**Author’s response:** We have considered. In multilevel analysis study, the determinant factors are usually identified by model III (a model fitted for both individual and community level variables). Model III shows the main findings and is used for interpretation and discussion while other models including model II are used for model comparison because of cluster/hierarchical nature of DHS data. Due to the cross-sectional nature of the study, variables that are aggregated at the community level might potentially mask within-community variations and might not show the real association between community variable and outcome variable. Thank you very much.

12. I also recommend you show the spatial distribution.

**Author’s response:** Thank you for your recommendation but it is difficult to construct the map because the geographical locations of the six selected countries are in different areas.

13. The manuscript contains several language issues that need to be addressed to improve clarity and readability, such as the Sentence Structure, Voice, Verb Tense, and Punctuation.

**Author’s response:** Thank you very much; we have made significant editorial and language corrections throughout the whole section of the manuscript
