# Supplementary material for: Determinants of formula feeding among mothers with infants and young children in six Sub Sahara African countries: Multilevel analysis of data from demographic and health survey
Source: PLoS One. 2024 Dec 26;19(12):e0311945. doi: 10.1371/journal.pone.0311945 (PMC11670973; doi:10.1371/journal.pone.0311945)
Supplement: Attachment — Submitted filename: Response to reviewer comments R2.docx [file pone.0311945.s004.docx]

**Response to reviewer comments**

**Reviewer #1:** Comments on statistical significance between institutional delivery, maternal educational level and media coverage has not been addressed properly. Do not just link it with your logic; add scientific reference contrary to your findings.

**Author’s response:** Dear reviewer, thank you very much for your insightful feedback. You are correct that statistical significance does not always fully capture the complexity of real-world outcomes. We have made amendments on the discussion section of the manuscript. While factors such as maternal education, institutional delivery, and media exposure were statistically linked to bottle feeding in our study, we acknowledge that these associations may not directly translate into practical or policy-related justifications without considering broader contextual factors. For instance, the promotion of institutional delivery may aim to improve maternal and child health outcomes, but it does not necessarily mitigate the influence of cultural practices or socioeconomic conditions that drive formula feeding. This underscores the need for a nuanced interpretation of findings that integrates both statistical significance and the broader public health context, as emphasized in similar studies. Other similar studies also proved our finding cited in the discussion section of the manuscript.

Furthermore, causal inference about the linkage between formula feeding and the above factors would require additional country-specific qualitative and quantitative studies. We have stated recommendation for future researchers on the discussion section of the manuscript.

**Reviewer #2:** Thank you for the detailed updates to the manuscript. I have no issues to accept the manuscript in its current form.

**Author’s response:** Dear reviewer, thank you very much
